# Mechanics and Microstructure of the Atrioventricular Heart Valve Chordae Tendineae: A Review

**DOI:** 10.3390/bioengineering7010025

**Published:** 2020-03-12

**Authors:** Colton J. Ross, Junnan Zheng, Liang Ma, Yi Wu, Chung-Hao Lee

**Affiliations:** 1 Biomechanics and Biomaterials Design Laboratory, School of Aerospace and Mechanical Engineering, The University of Oklahoma, Norman, OK 73019, USA; cjross@ou.edu (C.J.R.); yiwu@ou.edu (Y.W.); 2 Department of Cardiovascular Surgery, The First Affiliated Hospital of Zhejiang University, Hangzhou 310058, China; zhengjunnan@zju.edu.cn (J.Z.); ml1402@zju.edu.cn (L.M.); 3 Institute for Biomedical Engineering, Science and Technology (IBEST), The University of Oklahoma, Norman, OK 73019, USA

**Keywords:** the mitral valve, the tricuspid valve, collagen fiber architecture, glycosaminoglycan, uniaxial mechanical testing, in-vitro flow loops, polarized spatial frequency domain imaging

## Abstract

The atrioventricular heart valves (AHVs) are responsible for directing unidirectional blood flow through the heart by properly opening and closing the valve leaflets, which are supported in their function by the chordae tendineae and the papillary muscles. Specifically, the chordae tendineae are critical to distributing forces during systolic closure from the leaflets to the papillary muscles, preventing leaflet prolapse and consequent regurgitation. Current therapies for chordae failure have issues of disease recurrence or suboptimal treatment outcomes. To improve those therapies, researchers have sought to better understand the mechanics and microstructure of the chordae tendineae of the AHVs. The intricate structures of the chordae tendineae have become of increasing interest in recent literature, and there are several key findings that have not been comprehensively summarized in one review. Therefore, in this review paper, we will provide a summary of the current state of biomechanical and microstructural characterizations of the chordae tendineae, and also discuss perspectives for future studies that will aid in a better understanding of the tissue mechanics–microstructure linking of the AHVs’ chordae tendineae, and thereby improve the therapeutics for heart valve diseases caused by chordae failures.

## 1. Introduction

The atrioventricular heart valves (AHVs) regulate the unidirectional flow of blood through the heart chambers by the cyclic opening and closing of soft tissue leaflets. These leaflets are supported in their functions by the chordae tendineae, which attach to the papillary muscles. The chordae structures help to distribute load to the papillary muscles during systolic closure and prevent leaflet flail into the atria. In the case of chordae failure, such as elongation, rupture, thickening, retraction, or calcification, the result is a backflow of blood from the ventricle into the atria during the valve’s systolic closure–known as AHV regurgitation [1,2]. Uncontrolled severe AHV regurgitation can ultimately lead to left or right heart failure and yield poor outcome including potential years of life lost.

Chordae rupture affects the overall atrioventricular heart valve behaviors and can be a primary cause of valve regurgitation [3,4,5]. Khoiy et al. (2018) used an in vitro valve apparatus to observe changes in the tricuspid valve closure after induced chordae rupture, and found that ruptured chordae caused up to an 8.8% dilation of the annulus and consequent regurgitation [6]. In another in vitro study for the mitral valve, it was found that a significantly lower ventricular pressure was required to cause leaflet prolapse caused by chordae rupture [7]. Chordae failure plays a major role in valve regurgitation, however current surgical treatments have their pros and cons.

The major surgical treatments for chordae failure include shortening, transposition, and replacement. Chordal shortening involves making an incision in the papillary muscles and using a suture to pull a portion of the elongated chordae into the incised area [8]. The shortening method is undesirable, however, as it has a significantly lower freedom from recurrent mitral regurgitation as compared to chordal transposition (74 ± 9% and 96 ± 2%, respectively) [9]. Further, chordal shortening was also found to be inferior to chordal replacement, with the shortened chordae being more vulnerable to post-operation chordae rupture [10]. Chordal transposition, on the other hand, is performed by either attaching a portion of leaflet with chordae attachment from a non-prolapsing leaflet to a prolapsing leaflet, or by resection of the prolapsing leaflet’s chordal attachments. This technique also has drawbacks. For example, there is an approximate 13% recurrence rate of mild regurgitation in the long-term after mitral valve repair [11]. Chordal replacement is the current standard for chordae repair, in which the ruptured chordae is replaced with a synthetic material such as expanded polytetrafluoroethylene (ePTFE) sutures [10,12]. Despite the prevalence of the procedure, there are still issues such as elongation of the synthetic chordae, rupture of the native chordae, calcification, or recurrent prolapse potentially caused by an elastic modulus higher than that of the native chordae [13,14,15,16]. Various treatment methods for failed chordae tendineae can be improved and a better understanding of AHV diseases can be facilitated by studying the microstructure and mechanics of the native chordae tendineae. A more comprehensive understanding of the chordae tendineae will be especially beneficial to the refinement of AHV computational models and tissue engineering of chordae tendineae.

In this review paper, we will summarize the recent works in the microstructural and mechanical characterizations of the AHV chordae tendineae, as such a thorough review article is very limited in previous literature. In addition, we will also give some commentary remarks regarding the state of the art of research and perspective for future studies that would be beneficial to further the chordae biomechanics research field.

## 2. Overview on the Anatomy and Morphology of the AHV Chordae Tendineae

**Figure 1:**
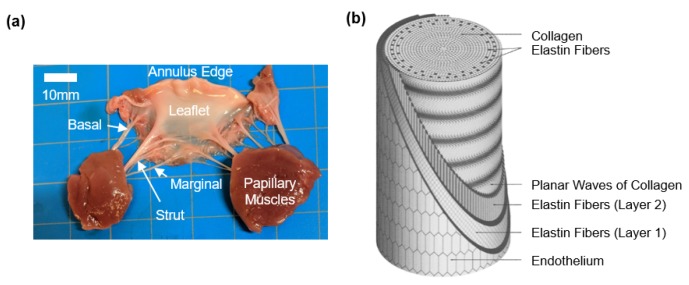
(**a**) An excised porcine mitral valve anterior leaflet with basal, strut, and marginal chordae tendineae. (**b**) Schematic of the microstructural components of the chordae tendineae (image from Millington-Sanders et al. [22] with permission from Wiley Global).

Chordae attaching to the leaflets are considered as true chordae, while those attaching elsewhere, such as the ventricular wall, are considered false chordae [17]. For this review, the focus will be on the true chordae, and any discussions henceforth are to be considered as referring to true chordae tendineae. Generally, chordae originate from the papillary muscle as a singular strand, which then either remains straight or branches into fan shapes prior to a leaflet insertion [17]. The chordae tendineae can be more specifically classified based on their respective insertion region to the leaflet: *basal chordae* attaching to the leaflet base; *marginal chordae* attaching to the leaflet free edge; and the notably-thicker *strut chordae* anchoring at the central belly region of the AHV leaflet (Figure 1a). Some differences exist between the chordae subsets. First, the strut chordae are the most critical subset for bearing load and are of special focus in the previous literature [18,19,20]. Secondly, in the tricuspid valve (TV), researchers have reported the marginal and basal chordae to be of a similar thickness. Thirdly, thicknesses of the chordae can also vary based on their leaflet of attachment, such as the mitral valve (MV) anterior leaflet chordae being thicker than the MV posterior leaflet chordae [21].

The number of chordae in the human, porcine, and ovine atrioventricular valve specimens have been analyzed in previous studies. Lam et al. and Silver et al. studied the human MV and TV, respectively, and reported an average of 25 chordae per valve [18,19]. Interestingly, De Almeida et al. noted that fetal hearts had fewer connective tissues (CT) and that there were proportionally fewer “muscular” (thicker, muscular texture) chordae as compared to the adult heart (reflected in both the MV and the TV) [23]. In comparison, we found in our previous study an average of 30 chordae in porcine MV and 35 chordae in the porcine TV, and an average of 15 and 24 chordae in ovine MV and TV, respectively [24]. These differences in the chordae anatomical features should be considered when discussing findings of porcine or ovine chordae studies and their translations to human heart anatomy.

It is worth noting that the different subsets of chordae (marginal, basal, and strut) are present in human, porcine, and ovine hearts but that the quantity can vary. However, to the authors’ knowledge, there is no study on quantifying the respective number of each chordae subset within the human, porcine, or ovine AHVs. Such important anatomical investigations are the first step to gaining insight to the chordae tendineae morphology, and further studies would be beneficial towards developing AHV computational models.

## 3. Chordae Tendineae Microstructure

### 3.1. Microstructures of Human AHV Chordae Tendineae

**Figure 2:**
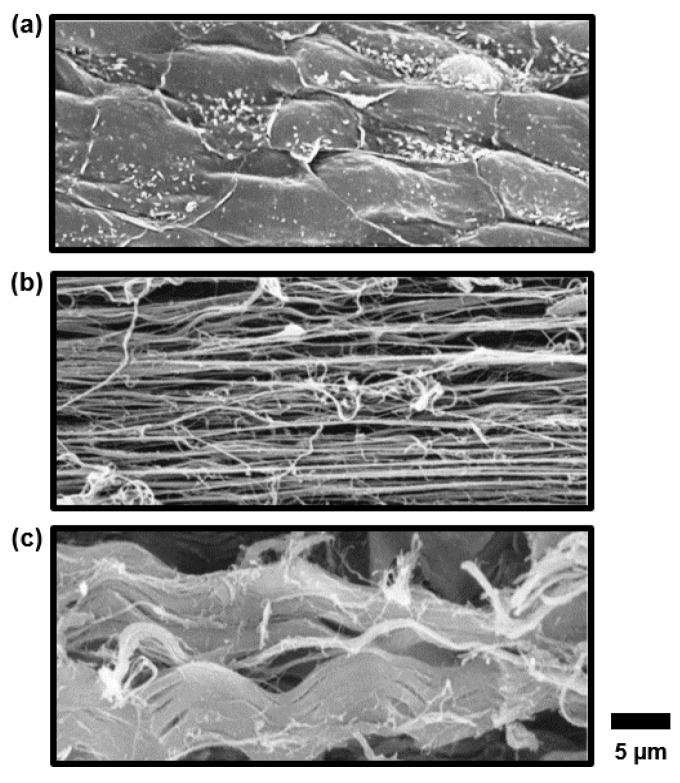
Scanning electron microscope (SEM) images of human mitral valve (MV) chordae tendineae, demonstrating (**a**) a layer of endothelial cells, (**b**) elastin fibers, and (**c**) undulated collagen fibers (image from Millington-Sanders et al. [22] with permission from Wiley Global).

**Figure 3:**
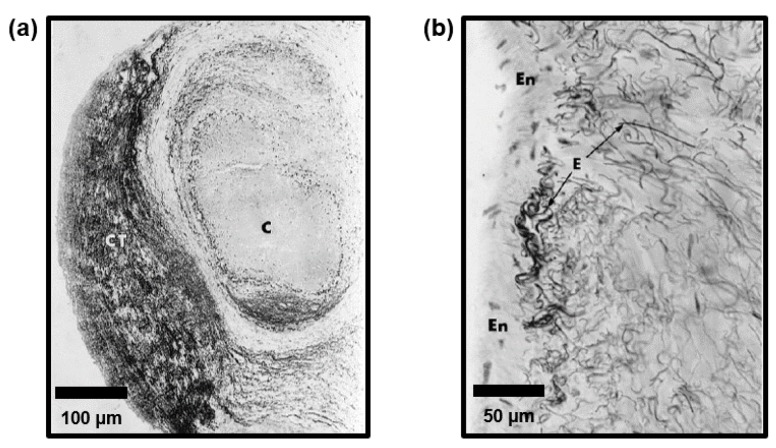
(**a**) A micrograph of a cross section of the chordae tendineae showing the collagen core (C) and the connective tissues (CT). (**b**) Within the connective tissue, a disorganized network of elastin fibers (E) and endothelium (En) exists (images from Millington-Sanders et al. [22] with permission from Wiley Global).

Regarding the chordae microstructure, a previous study by Fenoglio et al. showed that the chordae tendineae of the human mitral valve are composed of a core of collagen fibers surrounded by an elastin sheath [25]. Lim and Boughner (1977) took a closer look at the human MV chordae microstructure and described two forms of collagen: (i) a mostly straight, dense, collagen fiber core and (ii) widely spaced collagen fibers that wrap around the straight collagen fiber core with some angle of alignment against the primary axis [26]. In a later study, Lim’s research group used scanning electron microscopy (SEM) and transmission electron microscopy (TEM) to analyze the human TV chordae and found that the TV chordae were similar to the MV chordae, but that the TV chordae had a greater collagen fiber density and a smaller fibral diameter, owing to a lower force load experienced by the TV [27]. In later years, Millington-Sanders et al. investigated the microstructure of human MV chordae via SEM and light microscopy, and found an intricate layered structure (from outermost to innermost, Figure 1b, Figure 2 and Figure 3): (i) a layer of endothelial cells, (ii) an elastin sheath with fibers oriented at inclined angles with respect to the longitudinal axis, (iii) a longitudinally oriented elastin sheath, (iv) undulated collagen fibers aligned circumferentially, and (v) a core of straight collagen fibers with sparsely dispersed longitudinal elastic fibers [22].

### 3.2. Effects of Disease on Human AHV Chordae Tendineae Microstructure

Other research groups have studied the effects of disease on the chordae microstructures. Grande-Allen et al. [28] studied myxomatous human mitral valve chords of the posterior leaflet, with their key findings summarized as follows: (i) water contents of chordae was higher in valves with unileaflet and bileaflet prolapse relative to normal chordae; (ii) myxomatous valve chordae had significantly higher glycosaminoglycan (GAG) contents than the normal (healthy) chordae; and (iii) the relative increases in the GAG contents of myxomatous valve chordae were higher than those observed in the valve leaflets, suggesting that the disease might have a greater influence on the chordae biochemistry. Other researchers have also sought to understand the effects of a floppy mitral valve on the chordae. Several primary findings include: (i) floppy mitral valve chordae corresponded to greater collagen alterations and acid mucopolysaccharide accumulations (i.e., a proteoglycan that can contribute to calcification in severe cases) [29,30,31]; (ii) the increased proteoglycan contents could play a role in the degradation or defective formation of elastin and collagen; and (iii) the floppy mitral valve chordae had a disrupted collagen fiber core and surface fibrosis [32,33,34]. Lis et al. [33] also investigated the rheumatic (inflamed) chordae and found thickened collagen cores and minimal surface fibrosis.

### 3.3. Comparisons of the Chordae Microstructures Between Different Species

Due to the challenges in obtaining human chordae, porcine chordae have been widely used as a comparative model owing to their similarities to the human heart. However, differences have been found [35,36]. To elaborate, Ritchie et al. (2005, 2006) histologically examined porcine MV chordae, and they did not observe the distinct elastin layer as found by Millington-Sanders et al. for the human MV chordae [22,37]. Ritchie et al., however, did observe blood vessels in the chordae, supporting earlier findings by Duran and Gunning in fetal calf hearts [38]. The translation to human chordae structures should not be assumed, but the finding is interesting, as it describes another subtle role of the chordae tendineae—a structure through which nutrients can be provided to the valve leaflets. Liao et al. (2009) also noted a difference between the porcine and human MV chordae, finding the collagen fibers to be a 3D, wavy structure, as opposed to the planar, undulated collagen fibers observed in the human chordae [39]. Comprehensive studies on the human chordae microstructure using more modernized techniques would be valuable for confirmation of the similarities between the various species.

### 3.4. Comparisons of the Microstructures Between Chordae Subsets

The three subsets of chordae tendineae have varied microstructures. In a study of porcine MV chordae, it was found that the marginal chordae have a larger fiber density and a smaller fibral diameter than the basal or strut chords [40]. Liao and Vesely (2004) analyzed the GAG contents of the chordae and found GAG concentrations in a decreasing order: marginal, basal, and strut [41]. They hypothesized that the observed varied GAG concentrations among chordae subsets may factor into the differences in the mechanical properties and the structural functions, such as GAG-mediated fibril-to-fibril linkage.

### 3.5. Microstructures of the Chordae Insertion Regions

Another integral part of the chordae’s microstructure is the chordae-leaflet insertion region, where the highly aligned collagen fibers of the chordae transition into the more complex collagen fiber architecture of the leaflets. Chen et al. (2014) studied the strut chordae-leaflet insertions of porcine mitral valves and found that collagen fibers in the leaflet closer to the annulus were more circumferentially aligned, and that those collagen fibers became more radially aligned and uniform approaching the leaflet-chordae transition [42]. The chordae-papillary muscle insertion region has also been examined, and it was found that both human and porcine chordae exhibited a smooth, continuous endocardium endothelium between the chordae and papillary muscles [43]. However, differences were observed in the collagen fiber connection to the muscle, with human hearts containing an organized cross-network while porcine hearts were more random in architecture.

### 3.6. Microstructures of the Artificial Chordae

Artificial chordae tendineae, generally made of expanded polytetrafluoroethylene, are one of the primary treatment options for failed chordae [44]. The ePTFE sutures are composed of a high molecular weight compound of carbon and fluorine, and made porous with a surface charge that reduces thrombogenicity [16]. When implanted in the body, the artificial chordae will, over time, become fully encapsulated with dense fibrous tissue, covered by a layer of endothelial cells, while maintaining normal mechanical function [45]. However, due to the porous nature of the suture, the artificial chordae can become calcified, leading to eventual rupture [16,46]. Future research efforts may be devoted to refining the microstructure of the artificial chordae to reduce the chances of disease-based failure.

## 4. Tissue Mechanics of the Chordae Tendineae

**Figure 4:**
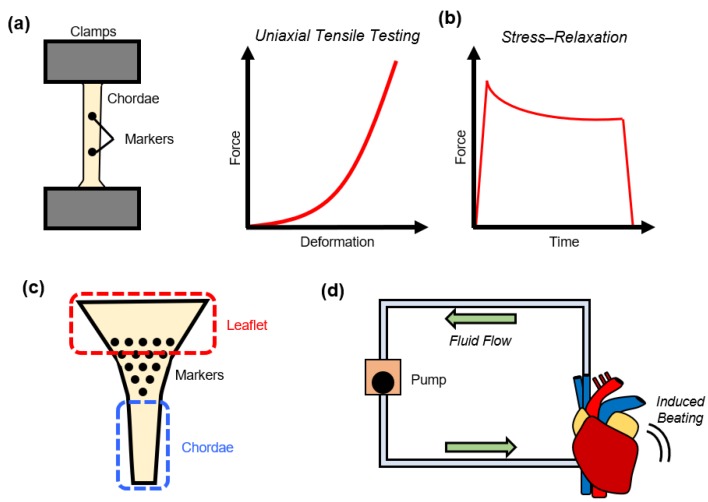
Mechanical characterizations of the atrioventricular heart valve (AHV) chordae tendineae through: (**a**) uniaxial mechanical testing, (**b**) stress-relaxation testing, (**c**) chordae-leaflet insertion region deformation tracking, and (**d**) an in vitro heart simulating flow loop.

In addition to gaining an understanding of the chordae’s microstructure and morphology, research efforts have been made to investigate the mechanical properties of the chordae tendineae. Studies of the chordae biomechanics include: (i) uniaxial tensile testing; (ii) stress-relaxation testing; (iii) chordae-leaflet insertion region testing; and (iv) in vitro flow loop testing (Figure 4).

### 4.1. Uniaxial Mechanical Testing of the Chordae Tendineae

The most prevalent method for investigating the chordae mechanics in previous literature is through uniaxial tensile testing. Generally, in these studies chordae were fully separated from their valve attachments and placed into a hydraulic uniaxial tensile testing machine (Figure 4a). Then, the chordae specimens were preconditioned, followed by either cyclic force loading and unloading to and from a target load, or loading until tissue rupture.

#### 4.1.1. Uniaxial Tensile Characterizations of Human Chordae Tendineae

One of the earliest documented studies using this method is from Lim and Boughner (1975). They applied monotonically increased uniaxial loading to human MV chordae until rupture. The three primary findings from their chordae characterization study were: (i) chordae were less extensible at an increasing strain rate, (ii) larger cross-sectional areas corresponded to a lower extensibility, and (iii) chordae ruptured at a strain of 21.4% and a stress of 3.1 × 10^8^ dyne/cm^2^ [47]. The rupture stress was consistent with a previous study, whereas the rupture strain varied (21.4% vs. 40%) [48]. In a subsequent study by Lim and Boughner (1976), human MV chordae were treated with enzymes to remove the elastin sheath prior to uniaxial mechanical testing. It was found that the removal of the elastin sheath did not significantly affect the elastic response of the chordae tissue. However the inter-specimen variability may overlap with the variation of their quantified mechanical results, as different specimens were used for control and treatment groups [49]. Future investigations using a unified testing scheme would be beneficial, where a specimen is tested as a control specimen, treated to remove the elastin sheath, and tested again for a direct comparison of the changes in the tissue mechanics due to elastin depletion. Lim (1980) also conducted mechanical testing for the TV chordae and found that the TV chordae were less extensible than their MV counterparts [27]. A potential shortcoming of these studies, however, was that machine cross-head displacements were used for quantifying tissue stretch, as opposed to calculating tissue strains using fiducial markers. Other researchers have also characterized human MV chordae using the more accurate fiducial marker approach, such as Zuo et al., for investigating the age-dependent changes in the chordae mechanical properties [21]. They observed stiffer and less extensible chordae as compared to earlier human chordae studies. Differences in the tissue mechanics could be possibly attributed to the use of the marker-based quantification of tissue strains, or potential variations in the patient age. Clark (1973) also studied the effects of freezing on human chordae tensile characteristics and observed that freezer storage resulted in a stiffer mechanical response [50].

#### 4.1.2. Effects of Disease on the Tensile Characteristics of Human Chordae Tendineae

Other researchers have studied the effects of disease on the human chordae tendineae’s uniaxial tensile behaviors. Barber et al. studied chordae from myxomatous valves and observed that diseased chordae had significantly lower moduli (40.4 ± 10.2 vs. 132 ± 15 MPa) and failure stresses (6.0 ± 0.6 MPa vs. 25.7 ± 1.8 MPa) than healthy chordae, but the extensibility and failure strain were similar [51]. Lim et al. also analyzed myxomatous chordae of the tricuspid valve and found higher extensibilities, lower rupture stresses, and similar rupture strains in diseased specimens compared to healthy specimens [52]. Differences in the myxomatous chordae behaviors between the AHVs could be attributed to differences in the chordae morphology, however more detailed studies are necessary. To better understand the effects of calcification, Casado et al. analyzed calcified marginal chordae by means of quasi-static tensile testing and observed that the diseased chordae were three to seven times more compliant than normal chordae [53].

#### 4.1.3. Mechanical Characterizations of Porcine Chordae Tendineae

**Figure 5:**
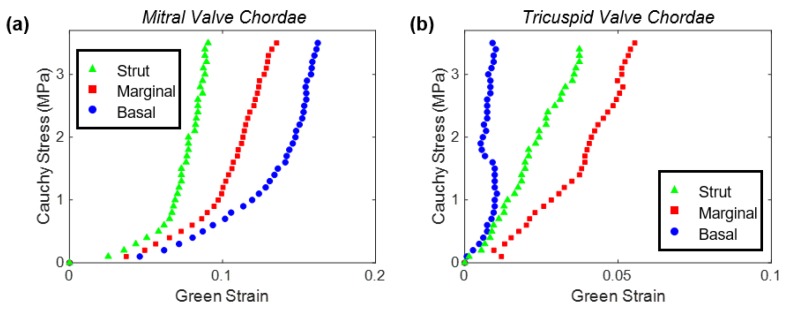
Results from the uniaxial mechanical testing of the strut, marginal, and basal chordae tendineae from: (**a**) the mitral valve anterior leaflet, and (**b**) the tricuspid valve anterior leaflet. (Plots reformatted from the data reported in Pokutta-Paskaleva et al. [55]).

There has also been extensive investigations of chordae mechanics through the study of porcine chordae tendineae. For example, Ritchie et al. (2006) uniaxially tested porcine chordae with their primary finding being that quantifying stretch using machine cross-head displacements corresponded to different extensibilities compared to using graphite markers [54]. Pokutta-Paskaleva et al. characterized porcine MV and TV chordae using the marker-based approach and observed that: (i) the strut chordae were stiffer than the marginal and basal chordae; (ii) the basal chordae had greater extensibilities than the marginal chordae; (iii) the MV chordae were stiffer than their TV counterparts; and (iv) the chordae attaching to the TV septal leaflet were more extensible than the chordae attaching to the other two TV leaflets (Figure 5) [55]. Sedransk et al. characterized the rupture of the porcine MV chordae using a tensile testing device and found that the marginal chordae ruptured at 68% less load and 28% less strain than the basal chordae [56]. They also found that the chordae from the MV posterior leaflet ruptured at 43% less load and 22% less strain than ones from the MV anterior leaflet.

Unique mechanical–morphological findings from other studies on the chordae include: (i) decellularized and glutaraldehyde cross-linked chordae (a method for replacing human chordae with porcine chordae) had a longer fatigue life and a lower creep rate than the native chordae [57], (ii) fatigue-induced micro-cracks in the collagen structures can cause increased creep behaviors [58], and (iii) glutaraldehyde-fixed chordae have decreased storage moduli compared to native structures [59].

It is worthwhile to note that while there are many studies characterizing MV chordae, the information regarding TV chordae mechanics is limited, and future investigations are warranted. In our lab, we made a recent contribution to the understanding of MV and TV strut chordae mechanics under a novel uniaxial tensile testing scheme [60]. In this study, we performed mechanical characterizations of the chordae tendineae where the attachment regions were preserved, resulting in a leaflet-chordae-papillary muscle entity with full planar deformation of the insertion regions. From this unique experimental setting, we observed different chordae mechanics than those reported in previous literature, which may be due to distributions of stress and deformations from the chordae to the leaflet and papillary muscle structures. A similar preliminary study (n = 1 strut chordae for each valve) was recently performed, but in this study the authors bisected the entity such that only a leaflet-chordae segment and a chordae-papillary muscle segment were tested [61]. Reported results from the uniaxial mechanical testing of chordae tendineae are summarized in Table 1.

### 4.2. Stress-Relaxation Testing of the Chordae Tendineae

To supplement the information on the chordae uniaxial tensile characteristics, Liao and Vesely (2004) conducted stress-relaxation testing of porcine MV chordae [41]. In the testing, they displaced tissues to the deformation associated with an initial 150 g load and observed the stress-relaxation behaviors over 100 seconds (Figure 4b). They observed strut chordae to have the fastest and greatest relaxation behavior (49.1 ± 5.4%), followed by the basal chordae (42.4 ± 8.3%), and then the marginal chordae (33.2 ± 4.7%). They proposed that the differences could be attributed to differences in the GAG contents, with chordae subsets containing fewer GAGs corresponding to greater relaxation behaviors. In other words, the marginal chordae had the largest GAG concentrations, and the smallest relaxation behaviors, whereas the strut chordae had the greatest relaxation behavior, but the lowest GAG contents. In previous studies of the valve leaflets, in contrast, fewer GAG contents corresponded to less stress relaxation [63,64]. The differences in the trends between the relaxation behaviors and the GAG contents may be due to the GAGs serving different roles in the mechanical behaviors for the leaflets or the chordae. Future studies are warranted to better elucidate the GAG contributions to mechanical behaviors in the chordae, such as through stress-relaxation testing of the tissues before and after enzymatic removal of the GAGs. Additionally, stress-relaxation studies could be performed on the TV chordae to better understand the differences in chordae mechanics for each AHV.

### 4.3. Load-Dependent Collagen Fiber Architecture of the Chordae-Leaflet Insertion Region

There are few studies focused on the mechanics of the chordae-leaflet insertion area. Padala et al. performed such investigations on porcine MV strut chordae using an in vitro flow loop by tracking an array of markers across the surface of the insertion region (Figure 4c) [65]. From this, they found that the edges of the insertion region stretched more than the center, which may be attributed to the chordae-leaflet transition region microstructure. Chen et al. (2004) also examined the porcine MV strut chordae region using a biaxial testing system [42]. In their testing they fixed the leaflet on three edges via suture hooks, and on the fourth edge attached the chordae to a string. They found that along the insertion region the radial extensibility decreased, and the stiffness increased. Another recent preliminary study was published to analyze the leaflet-chordae and papillary muscle-chordae insertion areas using X-ray diffraction [61]. Their preliminary finding was that the leaflet and papillary muscle insertions have a higher molecular strain than the rest of the chordae, suggesting those areas are more rupture vulnerable. Limitations of that study include: a relatively small sample size (n = 1 strut chordae for each valve), a very limited field of view, and the bisection of the chordae.

**Figure 6:**
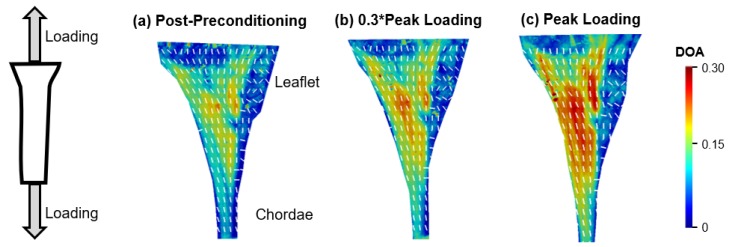
Quantifications of the load-dependent collagen fiber architecture of a representative porcine mitral valve strut chordae-leaflet insertion region using the polarized spatial frequency domain imaging (pSFDI) modality: (**a**) at the post-preconditioning configuration, (**b**) at intermediate loading (0.3 * peak loading), and (**c**) at the peaking loading. White, dashed lines denote the quantified collagen fiber orientation angles, and the colormaps show the degree of optical anisotropy (DOA)—a metric related to the degree of alignment for the underlying collage fiber networks (a warmer color denoting a better aligned collagen fiber network).

Extensive studies on the chordae-leaflet insertion have not yet to be performed for TV chordae tissues. In our lab, we recently completed a pilot study by using the polarized spatial frequency domain imaging (pSFDI) modality [66,67] to analyze the load-dependent collagen fiber orientations of the strut chordae insertions of porcine MV and TV anterior leaflets (MVAL and TVAL). Briefly, in the study we loaded leaflet-chordae-papillary muscle entities to physiologically representative loading (MVAL: 1.4 N and TVAL: 1.2 N), and pSFDI was performed to quantify the collagen fiber architecture at various states of the force-deformation curve (Figure 6).

### 4.4. In Vitro Flow Loop Testing of Chordae Tendineae

The use of in vitro flow loops is another experimental way to quantify the mechanics of the chordae tendineae (Figure 4d). Ritchie et al. (2006) used the Georgia Tech left heart simulator in conjunction with a marker-tracking approach to analyze the mechanical responses of porcine MV strut chordae [54]. They found that the chordae experienced a strain rate of 75.3 ± 3.43% during systolic closure and a strain rate of −54.8 ± −56.6% during diastolic opening. Furthermore, there was a constant plateau of the chordae strain between 3.75% and 4.29% during valve closure, indicating a minimal creeping response. Padala et al. used the same in vitro flow loop to quantify the tissue mechanics of the MV strut chordae-leaflet insertion (see Section 4.3) [65]. To the best of our knowledge, no studies have been conducted using in vitro flow loop methods for TV chordae—such future studies would be beneficial to make the connection between the chordae’s mechanical behaviors and the overall function of the tricuspid valve.

### 4.5. Mechanics of Artificial Chordae

The ePTFE artificial chordae used in chordae replacement have been analyzed for their mechanical properties, and how they compare to native chordae mechanics. In general, it has been found that ePTFE chordae do not have similar mechanical properties to native chordae. In particular, Caimmi et al. [68] found that ePTFE chordae were of higher compliance than their native counterparts, and that the stiffness of ePTFE chordae increases with the length of the implant. These findings would be beneficial to the refinement of therapeutics to better emulate native structures.

## 5. Closing Remarks and Future Prospects

There are several studies on MV strut chordae, but there is limited information pertaining to other subsets of MV chordae, and even more limited information on TV chordae. It would be worthwhile to investigate these under-represented structures through either in vitro flow loop or extensive uniaxial mechanical testing procedures, including tensile tests and stress-relaxation. For example, it would be interesting to understand how the GAG contents of the chordae affect the stress-relaxation behaviors through enzymatic procedures (similar to a previous study from our lab on AHV leaflets [64]). In addition, the stress-relaxation behavior could be analyzed in connection to the amount of the mechanical force acting on the tissues [65]. It would also be useful to better understand the contributions of the elastin sheath to the chordae’s overall mechanical behaviors through testing the same tissues before and after sheath removal. Other useful information could be found through new microstructural quantification technologies, such as polarized spatial frequency domain imaging, allowing for an understanding of the load-dependent collagen fiber architecture, especially at the insertion regions [66,67,69].

Findings from these studies are all critical to the future development and refinement of computational models of AHVs. Some of these models employ one set of material properties for all chordae, not considering the differences in the tissue mechanics and the structure of various chordae subsets (i.e., basal, marginal, and strut) [70,71,72]. There are, however, some computational models which consider these subsets, providing richer and more realistic predictions of heart valve biomechanical function [73,74,75]. To further improve these models, Khalighi et al. developed a topological and geometric mapping technique for the chordae tendineae of the MV, including branching, probable leaflet insertions, and cross-sectional areas [76]. A similar mapping has been realized for TV chordae as well [77]. Later, Khalighi et al. also proposed a simplified model of MV chordae that is functionally equivalent and significantly reduces computational complexity [78]. Simulation tools such as these are critical to therapeutic refinement and patient-specific surgical planning [74,79,80,81], as can be found in the simulation of MV annuloplasty and its effects on the forces experienced by the chordae tendineae [82]. Moving forward, future studies could include modeling of the microstructure of the chordae tendineae (Figure 1, Figure 2 and Figure 3), and developing computational models of the under-investigated TV chordae [83]. Another study would be incorporating the properties of diseased chordae/valve leaflets to predict the effects of diseased conditions, such as valve calcification, on the hemodynamics and homeostasis of the atrioventricular heart valves. Computational models incorporating the chordae tendineae mechanics and microstructure information will be critical for applications such as the development of microstructurally informed constitutive models, models with collagen fiber recruitment and reorientation predictions, and the growth and remodeling framework.

In summary, there have been foundational strides towards a better understanding of the chordae tendineae of the AHVs. Specifically, the morphology and microstructure of the chordae have been well-defined for both human and porcine chordae, for each of the chordal subsets, and for the MV and the TV. However, there are discrepancies between different studies that require further and more systematic investigations. Currently, there are no standard protocols for investigating chordae mechanics or microstructure. Through a review of previous research efforts, future efforts may be better guided for more detail and greater consistency. Moreover, the tissue mechanics of porcine MV strut chordae have been well characterized, but future studies are warranted regarding the mechanics of TV chordae, as well as the linking of the tissue mechanics and microstructures for human chordae. Furthermore, there are limited investigations regarding the chordae-leaflet insertion region other than our preliminary results for porcine MV strut chordae. Through more comprehensive investigations such as those mentioned above, efforts towards improved therapies and treatment outcomes can be better informed.

## Figures and Tables

**Table 1:** Comparisons of the reported uniaxial mechanical testing results (tissue stretch versus Cauchy stress) of the chordae tendineae of the mitral valve anterior leaflet (MVAL) and tricuspid valve anterior leaflet (TVAL).

		**MVAL Strut**		**TVAL Strut**	
Study	Species	Tissue Stretch *λ* (−)	Cauchy Stress (MPa)	Tissue Stretch *λ* (−)	Cauchy Stress (MPa)
Pokutta-Paskaleva et al. (2019) [55]	porcine (n = not provided)	1.09	3.5	1.04	3.5
Ritchie et al. (2006) [54]	porcine (n = not provided)	1.05	0.89 to 1.18	–	–
Liao and Vesely (2003) [40]	porcine (n = 16)	1.16 ± 0.03 (mean ± SD)	0.75 ± 0.15 (mean ± SD)	–	–
Zuo et al. (2016) [21]	ovine (n = 18)	1.07 ± 0.08 (mean ± SD)	24 (mean ± SD)	–	–
Ross et al. (2020) [60]	porcine (n = 12)	1.03 ± 0.01 (mean ± SEM)	1.59 ± 0.16 (mean ± SEM)	1.02 ± 0.01 (mean ± SEM)	2.71 ± 0.10 (mean ± SEM)
		**MVAL Marginal**		**TVAL Marginal**	
Pokutta-Paskaleva et al. (2019) [55]	porcine (n = not provided)	1.13	3.5	1.05	3.5
Kunzelman and Cochran (1990) [62]	porcine (n = 31)	1.09	1.96 ± 0.20 (mean ± SEM)	–	–
Liao and Vesely (2003) [40]	porcine (n = 16)	1.04 ± 0.01 (mean ± SD)	5.22 ± 3.30 (mean ± SD)	–	–
		**MVAL Basal**		**TVAL Basal**	
Pokutta-Paskaleva et al. (2019) [55]	porcine (n = not provided)	1.15	3.5	1.01	3.5
Kunzelman and Cochran (1990) [62]	porcine (n = 29)	1.12	1.57 ± 0.05 (mean ± SEM)	–	–
Liao and Vesely (2003) [40]	porcine (n = 20)	1.08 ± 0.03 (mean ± SD)	2.41 ± 0.81 (mean ± SD)	–	–
